# Phytochemical Profiling and Therapeutic Potential of Thyme (*Thymus* spp.): A Medicinal Herb

**DOI:** 10.1002/fsn3.4563

**Published:** 2024-11-01

**Authors:** Marwa Waheed, Muhammad Bilal Hussain, Farhan Saeed, Muhammad Afzaal, Aftab Ahmed, Rushba Irfan, Noor Akram, Faiyaz Ahmed, Gebremichael Gebremedhin Hailu

**Affiliations:** ^1^ Department of Food Science Government College University Faisalabad Faisalabad Pakistan; ^2^ Department of Nutritional Sciences Government College University Faisalabad Faisalabad Pakistan; ^3^ Institute of Home Sciences, Faculty of Food, Nutrition & Home Sciences University of Agriculture Faisalabad Faisalabad Pakistan; ^4^ Food Safety & Biotechnology Lab, Department of Food Science Government College University Faisalabad Faisalabad Pakistan; ^5^ Department of Basic Health Sciences, College of Applied Medical Sciences Qassim University Buraydah Saudi Arabia; ^6^ Food Technology and Process Engineering Oda Bultum University Chiro Ethiopia

**Keywords:** anticarcinogenic, antimicrobial, antioxidant, medicinal plant, therapeutic, thyme, thymol herb

## Abstract

Thymol is a phenol monoterpene that is naturally derived from cymene and is an isomer of carvacrol. It constitutes a significant portion (10%–64%) of the essential oils found in thyme (*Thymus vulgaris* L., Lamiaceae), a medicinal plant renowned for its therapeutic properties. Wild thyme is native to the Mediterranean region and has been used in cooking and medicine for a long time. In contemporary contexts, both thymol and thyme offer diverse functional applications in the pharmaceutical, food, and cosmetic industries. Thymol has attracted scientific interest for its potential therapeutic applications in pharmaceuticals and nutraceuticals. Studies have explored its efficacy in treating respiratory, nervous, and cardiovascular disorders, highlighting its promising role in diverse therapeutic interventions. Additionally, this compound demonstrates antimicrobial, antioxidant, anticarcinogenic, anti‐inflammatory, and antispasmodic properties. It also shows potential as a growth enhancer and has immunomodulatory properties as well. Other discussed aspects include thymol toxicity, bioavailability, metabolism, and distribution in animals and humans. This review summarizes the most significant data regarding the beneficial effects of thyme bioactive compounds and their applications as a food preservative while taking into account the thyme plant extract and its essential oil.

## Graphical abstract

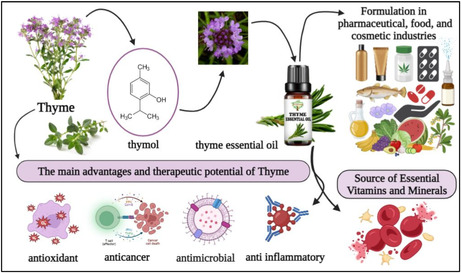

## 1 Introduction

Natural medicinal plants are being used in various industrialized and developing countries as a substitute for artificial or synthetic drugs. Traditional application of medicinal herbs suggests a significant cultural as well as historical usage, which is right for numerous foodstuffs available as “traditional herbal medicines” (Rai et al. 2023a). Nowadays, researchers are considering herbal plants because of their microbial action and bacterial resistance to antibiotics, which would otherwise lead to serious health issues if not managed properly. To meet healthcare demands, a large percentage of people rely on regular medical practitioners in various developing countries as they completely depend on medicinal plants (Paudel et al. 2024).

Herbal plants are considered an important source of novel medicines and a lot of contemporary treatments can be done ultimately from plant sources. Previous studies and research regarding herbal medicinal plants assist in understanding plant toxicity and also raise awareness about humans’ and animals’ protection from natural toxic substances (Hosseinzadeh et al. 2015). Aromatic and medicinal plants are included in the plants that have economic importance and have played a significant role in various human health conditions. The therapeutic importance of plants relies on the manifestation of elemental or nutritional composition and a group of phytochemical components that carry certain biological and physical activities in the human body (Castillo‐lópez et al. 2017). Among the medicinal and pharmacological natural sources, thyme is one of the biggest species of the genus “Thymus,” which belongs to the family *Lamiaceae*, which generally has notable blossoming plants with around 220 genera and 4000 species worldwide and can be used for culinary, cosmetic, and medicinal purposes (Mamadalieva et al. 2017). Thyme plant is also an important nectar source for honey bees and additionally, it is known as creeping thyme, mountain thyme, garden thyme, and common thyme (Khosravipour and Direkvand‐Moghadam 2016).

## 2 Origin of Thyme

Thyme is the name frequently used for various varieties of the species named “Thymus,” which is native to Asia and Europe. Among the varieties, common thyme is considered the major variety that is being utilized commonly for ornamental and flowering purposes. It is naturally present in the West Mediterranean region, which extends further to South‐Eastern Italy but can also be grown in different areas of the world (Nadiya, Yadav, and Anurag 2016). The name “thyme” was given first by the Greeks to the plant, meaning “to fumigate or disinfect with the fumes of particular plant,” because they utilized thyme as an important ingredient of the perfume industry, owing to its powerful aroma. Many others derived its name from two Greek words, Thyo (means perfume) or Thumus (means signifying courage) and for that reason, the thyme plant in prehistoric epochs became a great source of invigoration and courage (Dauqan and Abdullah 2017).

## 3 Plant Description

Thyme is a wild and woody perennial flowering shrub having extremely scented, gray–green and small‐sized leaves, and purple or pink flowers in a bunch that bloom from May to September with a distinctive fragrance. The strong aroma of thyme herb is felt because of thymol, and it is grown widely as an herb used in food products. It covers the ground and occasionally grows up to 40 cm with vertical as well as horizontal habits (Saleh et al. 2015). Stem of the plant becomes woody with age. It grows naturally as a bush or subshrub from 5 to 30 cm in height with fibrous roots as well as small, greenish‐gray leaves with narrow edges. Leaves are usually 2.5–5 mm long, have a very short petiole or sessile, and are rectangular to oval in shape. Moreover, above‐ground parts of this plant are used for the production of volatile oil through the vapor distillation process and dried thyme is available in the market for culinary purposes (Reddy et al. 2014).

Thyme develops very well for the duration of a dry, temperate, sunny, and hot climate, wherever these plants don't appear to be out of the sun. Various species of thyme grow and do best in rough and coarse soil that might have been proved to be inappropriate for quite a few other flowering plants. Fungal disease and root rot occur on thyme plants as a result of too many wet soil conditions. Thyme is usually planted in the spring season and needs sunlight to propagate or nurture up to its greatest possible limit. Thyme can be grown or spread by cuttings, dividing deep‐rooted segments of the plant, and also via seeds. In the case of planting via seeds, thyme had better be planted in early April or late March and seeds must be positioned around a half inch deep and 8–9 inches from each other. The roots could be replanted and divided from May to September. It grows better in lightweight and well‐drained soil with a pH range of 5.0–8.0 (Reddy et al. 2014). Various strategies might be present in the range from sun drying to high‐class automatic drying devices, but unfortunately, the use of the sun drying method indicates deprived volatile oil quality, whereas artificial drying techniques approve the higher product quality management. Leaves of thyme must be dried out at a temperature not more than 40°C to reduce the flavor loss through volatile oil evaporation and also to retain the green color of leaves (Reddy et al. 2017).

Dried parts, especially dried leaves (grayish‐green) of thyme, can be used for oleoresin production and essential oil. Thyme essential oil yields about 0.5%–1.5% when obtained via water or steam distillation of moderately dehydrated aerial parts of the plant and the resultant oil might be orange, brownish‐red, or grayish‐brown in color. Thyme has a pungent, warm, and slightly floral aroma with minty‐green hay‐like moldy and musty flavor (Borugă et al. 2014).

## 4 Varieties

**FIGURE 1:**
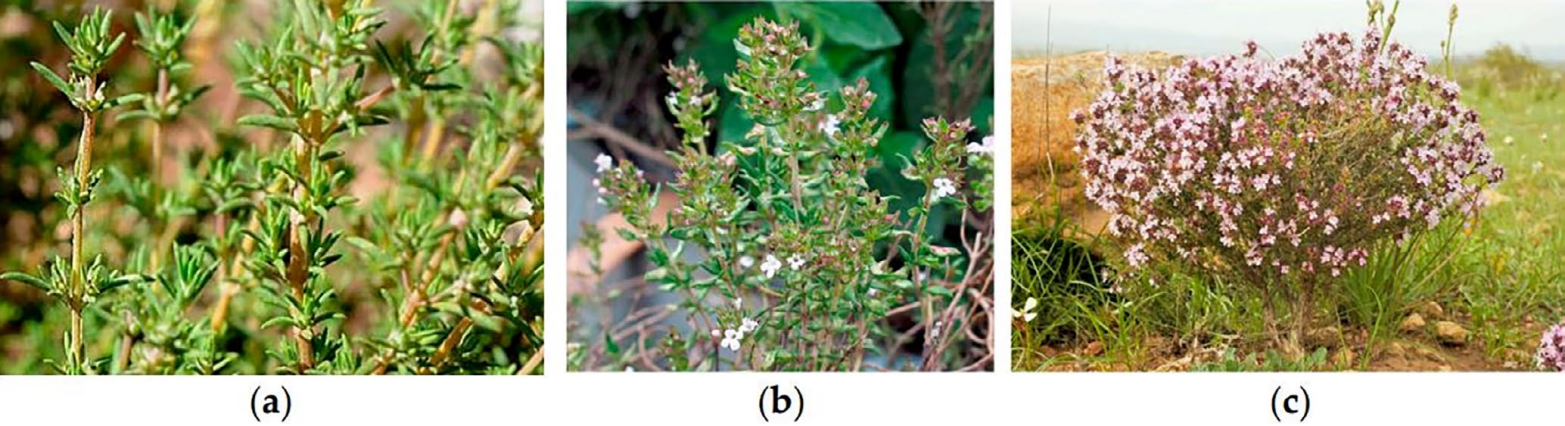
(a) *Thymus zygis*, (b)*Thymus vulgaris*, and (c)*Thymus hyemalis*.

The genus *Thymus* is one of the largest groups or members of the mint family that has about 215 species and some hybrids as well. Three main cultivars were grown‐up for typical consumption, such as variegated, narrow‐leaved, and broad‐leaved (Tabassi et al. 2015). The silver thyme (variegated) is more resistant than all other members of the mint family and has the strongest flavor. The narrow‐leaved variety has gray–green leaves and is more fragrant as compared with the broad‐leaved variety and recognized as German thyme. Lemon thyme has a lemon flavor and broader leaves with no curls at the margins. The most cultivated *Thymus* species used for medicinal, culinary, and essential oil extraction are *Thymus hyemalis, Thymus vulgaris*, *Thymus zygis*, *Thymus citriodorus*, and Varico. Varico is a vigorous cultivar with grayish‐blue leaves, which might have been propagated with seeds and yields about 50% of thymol, which accounts for more than 3% of essential oil (Tabassi et al. 2016) (Figure 1).

## 5 Nutritional Importance

Recently, consumer demands for beneficial food constituents have been increasing that may impart health benefits other than basic nourishment to the body. They not only represent a medium for the dissolution of functional components but also present a suitable way of consumption. The amazing health benefits of *Thymus vulgaris* can be credited to its high nutritional profile (Nanasombat, Thonglong, and Jitlakha 2015). Its nutrients in the following table are given below including proteins, carbohydrates, minerals, vitamins, aromatic substances, essential oil, carotenoids, phenolic antioxidants, flavonoids, and phenolic acids, which have medicinal and health‐promoting properties that are vital for optimum health. It has varied nutritional components and the nature of these components depends on the climatic conditions, variety, time of harvesting, nature of soil, and biological origin of thyme plant, as well as processing, packaging, and storage conditions (Tornuk et al. 2013).

Proteins, amino acids, carbohydrates, and mineral composition of thyme provide valuable information about its medicinal and nutritional quality (Thomas and Krishnakumari 2015). The determination of ash contents is important because it is considered as the measure of total number of minerals present in a food, while the determination of mineral content is a measure of the total amount of inorganic constituents contained by a food that further becomes the cause of pharmacological effect (Hameed and Hussain 2015). Proteins are important parameter for nutritional assessment of higher plants and participate virtually in different processes within the cell. Amino acids’ concentration as building blocks of protein includes threonine, proline, arginine, alanine, glycine, cysteine, valine, glutamic acid, and methionine in leaves and stem of thyme. Leaves contain high amount of methionine amino acid and low concentration observed for proline, whereas threonine was found in high concentration in stem and low concentration observed for glycine. Enzymes that catalyze many biological reactions are also proteins and these are vital to metabolism (Hasan, El‐Mehdawy, and Saad 2014).

Minerals, including both the macro and micro minerals, have been detected in thyme leaves, whether fresh or dried, and these include potassium (K), calcium (Ca), iron (Fe), manganese (Mn), copper (Cu), and magnesium (Mg) that are the major components in cells and extracellular fluids of an organism (Tomescu et al. 2015). Magnesium (Mg) and calcium (Ca) occur in higher amounts in medicinal plants of the genus *Thymus* Likewise, the average content of iron (Fe) in thyme is much higher than in the other plant materials. Potassium (K), because of being the key constituent of body fluids and cells, normalizes the blood pressure and heart rate. Manganese is considered as a cofactor for the activity of superoxide dismutase (SOD) enzyme. Iron (Fe) is involved as a significant component in the formation of red blood cells and plays an important role in the treatment of iron deficiency anemia (IDA) (Arceusz, Radecka, and Wesolowski 2010).

Thyme has strong composition of vitamins, particularly vitamin C (ascorbic acid) and vitamin A (retinol). Vitamin C helps the body to improve the resistance against infectious diseases as well as scavenge the pro‐inflammatory and damaging free radicals (Maggini et al. 2017). Vitamin A is known as antioxidant and fat‐soluble vitamin, which is essential for vision and is required for maintaining the skin and a healthy mucous membrane. Utilization of thyme rich in flavonoid components, for example, beta‐carotene and vitamin A aids in the protection from oral cavity and lung cancer (Sharangi and Guha 2013). Thyme is a good source of pyridoxine (B6) among the water‐soluble vitamins which with a serving of 100 g provides 27% (0.35 mg) of daily recommended intake of this vitamin. Vitamin B6 also promotes the maintenance of gamma‐aminobutyric acid (GABA) levels that is the main inhibitory neurotransmitter in brain and plays a role of stress buster. Several other vitamins are also being found in thyme herb including vitamin E, folic acid, and vitamin K (Dauqan and Abdullah 2017).

Carotenoids and chlorophylls are important plant pigments that are mostly polyphenolic because they are multiphenol‐containing molecules. Thyme contains large amount of chlorophyll (ubiquitous pigment), which is a detoxifier and an anticancer agent. Under certain conditions, beta‐carotene along with other pigments blocks free radical–mediated reactions and also reduces the occurrence of reactive oxygen species (ROS) in different conditions (El‐Qudah 2014).

Phenolic acids and flavonoid antioxidant compounds are also important constituents that play a significant role in nutritional composition and as a result, provide health benefits to the human beings. High‐performance liquid chromatographic (HPLC) technique for the analysis of extract of thyme leaves exhibited the incidence of methyl rosmarinate, cinnamic acid, rosmarinic acid, caffeic acid, phenolic acids, protocatechuic acid, and chlorogenic acid as these compounds are present in the group of phenolic acids and luteolin, quercetin, apigenin, ferulic acid, zeaxanthin, naringenin, and thymonin are verified as flavonoid compounds. The research experiments regarding the methanolic extract of thyme leaves reported about the higher antioxidant capacity of thyme than the natural and synthetic antioxidants like α‐tocopherol and BHA (Sharangi and Guha 2013).

Essential oil of thyme constitutes as a raw material in cosmetics and perfumery because of the distinct and characteristic fragrance (Grigore et al. 2010). Chemical compounds in essential oil are β‐myrcene, α‐pinene, sabinene, 1,8‐cineole, α‐terpineol, caryophyllene, carvacrol, thymol, apigenin, eugenol, p‐cymene, and rosmarinic acid that give their antioxidant and antimicrobial, anti‐inflammatory and antiseptic effects. Thymol is the main phenolic component of essential oil that is primarily responsible for its antioxidant activity and is identified as the most important constituent of thyme that is responsible for antifungal and antiseptic activities. Environmental factors, cultivation techniques, and genetic modification significantly affect thymol production in *Thymus* spp. High temperature and concentration of soil nutrient, particularly K and Ca, positively correlate with thymol level (Yavari et al. 2010). Genetic variation allows to adapt diverse habitats, which influence the phytochemical profiling (Németh‐Zámbori 2020). On the other hand, time of harvest can affect the essential oil components, because it varies throughout the growth period. Consequently, understanding the interplay between geographical factors and timing can provide valuable insights for optimizing cultivation practices aimed at maximizing thymol yield (Vaičiulytė, Ložienė, and Taraškevičius 2022).

Thyme also comprises other supplementary volatile oils, for example, geraniol, borneol, and carvacrol (Moghtader 2012). Fresh leaves of thyme indicated the total ORAC (oxygen radical absorbance capacity) of 27,426 μmol TE/100 g, which is the sign of having an elevated level of antioxidant activity (Brewer 2011) (Table 1).

**TABLE 1:** Nutritional profile of fresh *Thymus vulgaris* (Dauqan and Abdullah 2017).

Constituents	Value (per 100 g)	% RDA
Major constituents		
Carbohydrates	24.5 g	18%
Proteins	5.49 g	10%
Dietary fiber	13.99 g	37%
Total fat	1.67 g	8.4%
Vitamins		
Vitamin C	161 mg	266%
Vitamin A	4750 IU	158%
Thiamin (B1)	0.44 mg	3.8%
Riboflavin (B2)	0.470 mg	36%
Pantothenic acid (B5)	0.408 mg	8%
Niacin (B3)	1.825 mg	11%
Pyridoxine (B6)	0.346 mg	27%
Folic acid (B9)	44.94 μg	11%
Minerals		
Iron	17.46 mg	218%
Zinc	1.82 mg	16.49%
Potassium	610 mg	13.2%
Calcium	404 mg	40%
Sodium	8.96 mg	0.5%
Magnesium	161 mg	40%

## 6 Chemical Constituents of Thyme Essential Oil

Essential oil of thyme is known as a mixture of natural constituents that are present in relatively different quantities or concentrations. It has variant chemical compounds including monoterpene hydrocarbons (28.69%), oxygenated monoterpenes (56.53%), oxygenated sesquiterpenes (1.84%), and sesquiterpene hydrocarbons (5.04%). Among the constituents of essential oil, thymol (51.34%) is the major compound, whereas the amount of other components of essential oil comprise about less than 19% (Al‐Maqtari, Alghalibi, and Alhamzy 2011).

There are six chemotypes of thyme with different essential oil constituents. The main components in dried herbal preparations are carvacrol, thymol, linalool, β‐myrcene, terpinen‐4‐ol, p‐cymene, and γ‐terpinene that contribute up to 2.5% of essential oil and some other compounds appear to be present as glycosides. There are some most important chemical constituents of thyme (thymol, carvacrol, eugenol, linalool, apigenin, rosmarinic acid, and p‐cymene) that have their specific composition as well as biological action (Reddy et al. 2017).

**FIGURE 2:**
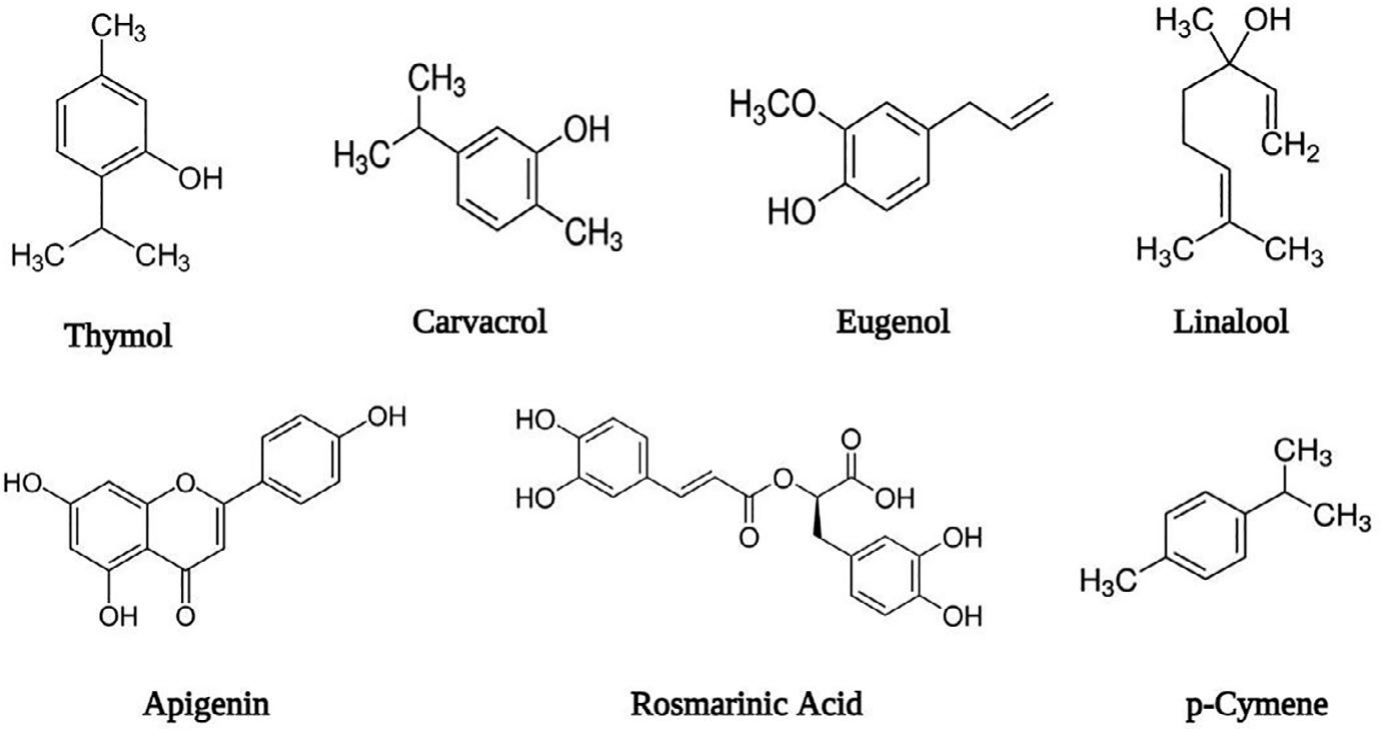
Structural representation of essential oil constituents (thymol, carvacrol, eugenol, linalool, apigenin, rosmarinic acid, and p‐cymene) (Dauqan and Abdullah 2017).

Monoterpene thymol (2‐isopropyl‐5‐methylphenol) is a white‐color crystalline phenolic compound and considered as the major constituent of thyme essential oil. It possesses aromatic sweet odor that also represents strong antioxidant, antiseptic, antifungal, and antibacterial properties (Venturini, Blanco, and Oria 2002). In essential oil of thyme as well as many other sweet‐smelling spices and herbs, carvacrol (5‐isopropyl‐2‐methylphenol) is an important monoterpene compound among the group of phenolics. Biological action of carvacrol has several effects that are reported as acetyl cholinesterase (AChE) inhibitory action, antimicrobial, anti‐inflammatory, and antithrombotic (Jamali et al. 2012) (Figure 2).

Eugenol [2‐methoxy‐4‐(2‐propenyl) phenol], which is present in several medicinal herbs, is used in dentistry from the past few decades. Due to analgesic properties, it acts as a hypothermic and anticonvulsive agent. Moreover, eugenol exhibits other different pharmacological properties including antianaphylactic, neuroprotective, and anti‐inflammatory (Javed et al. 2013).

Linalool is a monoterpenoid alcoholic compound and constitutes as a major component of volatile oil in thyme as well as present in a number of different aromatic plant species. Previous researches concluded that linalool compound has different biological activities, such as antioxidant, anesthetic, antiviral, antinociceptive, analgesic as well as anti‐inflammatory activities (Bagetta et al. 2010).

It is a flavonoid compound (4,5,7‐trihydroxyflavone). Several biological activities that are confirmed and exhibited by apigenin are anticarcinogenic, antiviral, antimutagenic, antioxidant, anti‐inflammatory, and antiprogression. Because of low intrinsic toxicity and prominent effects of apigenin on cancer and normal healthy cells, it gained particular importance in recent years as a health promising and useful agent as compared with other flavonoids that are similar in structure to that of apigenin (Liu et al. 2011).

Rosmarinic acid is another significant chemical compound 3‐(3,4‐dihydroxyphenyl‐lactic acid) present in thyme essential oil. It is astringent in taste with numerous therapeutic properties, for example, antimutagenic, antioxidant, antiallergic, anti‐inflammatory, and antiviral. Low toxicity can be exhibited after circulatory system administration and also when LD50 is used as 561 mg per kg body weight in rats and it can rapidly be eliminated from the blood circulation (Al‐Dhabi et al. 2014).

It is an aromatic compound in thyme plant, categorized as an alkylbenzene, and also relates to a monoterpenoid group. Its structural demonstration involves para‐substitution of isopropyl and methyl groups on a benzene ring. Various biological activities are shown by p‐cymene, which includes antinociceptive, antimicrobial, anti‐ inflammatory, antioxidant, anticancer, and anxiolytic activities. Current in vivo studies performed on experimental animal models determined the activity of antioxidant enzymes and oxidative stress reduction by p‐cymene (de Oliveira et al. 2015).

### 6.1 Extraction, Quantification, and Characterization of Thymol

Essential oil of thyme (thymol) can be extracted by using numerous techniques like steam distillation, hydrodistillation, and supercritical fluid extraction. It can also be extracted by ethyl acetate and cyclohexane in high‐performance thin‐layer chromatography (HPTLC). It can be quantified by analytical techniques that are gas chromatography–mass spectrometry (GC‐MS), HPLC, capillary electrochromatography (CEC), and liquid chromatography–tandem mass spectrometry (LC‐MS/MS) (Mottaleb, Meziani, and Islam 2019). The chemical composition of essential oil can be characterized by HPLC‐UV and GC‐MS, which identified carvacrol, p‐cymene, thymol, linalool, and γ‐terpinene as major components.

### 6.2 Standardization and Quality Control

Standardization and quality control measures for thymol are essential to ensure product efficacy, consistency, and safety. Standardization guarantees that every lot of thymol comprises active ingredients and manufacturers can lessen the risk of contamination with heavy metals, microbes, and pesticides by standardization of thymol products (Bhairam et al. 2013). Following the recognized standards, which helps companies to run into monitoring requirements and compliance with quality standards, for example, those set by the International Organization for Standardization (ISO), is essential for consumer trust and market access. Moreover, product quality, which can lead to improved customer satisfaction and loyalty, can also be enhanced by standardization. On the other hand, key aspects of quality control include the testing of purity and concentration of thymol. For this reason, chromatographic technique like HPTLC can be employed to measure the levels of thymol in various formulations, which assure us that they are formulated according to specified standards (Foudah et al. 2022).

### 6.3 Bioavailability and Metabolism of Thyme Essential Oil (Thymol)

Thymol is rapidly absorbed and then metabolized after oral administration in humans. Thymol sulfate and thymol glucuronide are the main metabolites, which are detected in urine as well as plasma (Pisarčíková et al. 2017). Other metabolites of thymol are p‐cymene derivatives and thymohydroquinone sulfate. As the free thymol form is not measurable in plasma, which is why it suggests extensive metabolism (Mason et al. 2017).

Few animal studies have demonstrated the fact in rabbits, where thymol is absorbed from the gastrointestinal (GI) tract and distributed to different tissues like liver, kidney, intestinal wall, and muscle. Furthermore, thymol is metabolized to sulfuric acid conjugates and glucuronic acid, which are then excreted in urine (Bacova et al. 2020). Whereas, thymol is metabolized to derivatives of p‐cymene and excreted in urine, in rats. It is also detected in the eggs of hens as well as Japanese quail which were fed thyme extract, and further transfers to palatable animal products. Thymol level and its metabolites vary according to the route, duration, and dose of administration. The above‐explained ADME (absorption, distribution, metabolism, and excretion) studies demonstrate that thymol can be easily absorbed, metabolized, and excreted from the body. The metabolic pathways include conjugation with sulfate and glucuronic acid (Al‐Harrasi et al. 2022).

## 7 Applications of Thyme

### 7.1 Thyme as a Natural Food Preservative

Preservatives are the constituents that originated from various natural and synthetic sources. Addition of preservatives in different food products and pharmaceuticals targets the spoilage that is instigated from undesirable changes at molecular level and from microbial development (Abdulmumeen, Risikat, and Sururah 2012). It acts as a constituent with the ability to obstruct and inhibit acidification and fermentation processes (Tzima et al. 2015). Thyme essential oils can also be suggested as a regular substitute of several natural compounds and as the alternate of synthetic or chemical preservatives. Because of the essential oil fraction, a number of herbs, plant extracts, and spices exhibit the preservative activities regarding their essential oil fraction (Negi 2012).

Preservative and antimicrobial activity of thyme gave it a rank among the most effective essential oils, as it is supported by numerous previous reports worldwide. Efficacy of thyme regarding antimicrobial action has been attributed mainly to phenolic compounds that are carvacrol, thymol, and p‐cymene present in volatile oil (Silva et al. 2013). Its essential oil is proved to act as a highly active ingredient in fungicides and could be utilized safely as a natural chemical ingredient that replaces artificial fungicides for the treatment of some plant fungal diseases.

Against the growth of microorganisms, researchers reported the antimicrobial activities of volatile compounds of plants in the Mediterranean region and thyme was among the one with inhibitory action. The most active inhibitory action is exhibited against *Lactobacillus plantarum*, *Brevibacterium linens*, and *Brochothrix thermosphacta* and is attributed to the powerful and active antibacterial constituents, such as thymol, phenols, and carvacrol (Nowak et al. 2013). Some previous scientific evidences reported that essential oil of thyme at low concentrations as (2%, 5%, and 8%) in a solution of propylene glycol and water as a creaming and emulsifying agent presents effective antioxidant activity when used for the Nile Tilapia fillets at refrigeration temperature (Albarracin et al. 2012).

The reduction of oxidative process in the fillets through the utilization of essential oil happened between the ranges of 5% and 96.5% and this range validates the efficiency of essential oil at lower level too. Some scientists in recent times evidenced the preservative and antibacterial effect of thyme to kill various foodborne microbial species, such as *Staphylococcus epidermidis*, *Pseudomonas aeruginosa*, *Staphylococcus aureus*, *Salmonella enterica*, *Enterococcus faecalis*, *Listeria monocytogenes*, *Enterococcus faecium*, *Escherichia coli*, *Bacillus cereus*, and *Clostridium perfringens* (Khalili et al. 2015; Silva et al. 2013). Previous scientific studies exhibited the antimicrobial potential of different thyme extract concentrations (0, 1000, and 2000 ppm) against *Pseudomonas fluorescens* and *Bacillus subtilis* suggested the utilization of thyme in edible coatings, which are considered beneficial so as to protect food products. The antimicrobial activity of thyme extract considered as improving the safety and preservation of foods (Ulbin‐Figlewicz, Zimoch, and Jarmoluk 2013).

### 7.2 Dairy Industry

In dairy industry, thyme essential oil exhibited the inhibition of pathogenic microorganisms like *S*. *aureus* and *L*. *monocytogenes*, which are linked with low‐ripened and fresh cheese. The starter coculture comprising *Lactobacillus cremoris* and *Lactobacillus lactis* was affected by essential oil at varying concentrations in cheese‐broth and in semisolid cheese model. Thus, essential oil doses are recommended for the inhibition or control of pathogenic bacteria in low‐ripened cheese among the fermented dairy products (Ballester‐Costa et al. 2013).

### 7.3 Baking Industry

In recent days, owing to the pronounced reducing and oxidizing properties, the thyme alcoholic extract holds the utilization and importance in frequent sections of food industry, particularly baking industry. Because of the active chemical constituents, effect of thyme extract on rheological and fermentation characteristics of dough is evaluated. In baking industry, the lost carbon dioxide (CO_2_) gas (mL) volume is not as much of that in the sample of dough because of the supplementary extracts of thyme with 1.0% concentration (Davidović et al. 2010). Results of these studies justified the alcoholic extracts of thyme herb as per additives in flour to encourage research on the utilization of natural raw materials in the baking industry as additives and also to enhance the dough's rheological characteristics. Resistance in dough takes place when thyme extracts (0.05% to 0.5%), L‐ascorbic acid (0.006%), and complex additives (0.3%) are taken into account, which in turn shows that thyme and the complex additives have a substantial role for firming up of gluten (Gonçalves et al. 2017).

In vitro action against molds and bacteria of encapsulated oil as well as free oil has been validated and after that, an appropriate quantitative assay was completed. Thyme‐free oil exhibited high activity against microorganisms and showed the values to be < 0.5 mg/mL. Additionally, the values of minimum inhibitory concentration (MIC) for encapsulated thyme oil were less than those of the free oil because of the protection from microorganisms as provided by the particle wall. The microparticles, which are formed by breaking down of the cell wall of microorganisms upon damage, are used for cake samples and give protection against the encapsulated oil volatilization and hence, increased the shelf life of the cake, up to 30 days without the utilization of artificial commercial preservatives (Gonçalves et al. 2017).

### 7.4 Meat Industry

Owing to the wide content of phenolic compounds along with the antioxidant and antimicrobial activities, thyme herb has gained considerable value (Bozin et al. 2006). Different research studies inspected the preservative qualities of thyme oil at concentrations from 1% to 2%, increased shelf life of meat as well as effect of thyme essential oil on microbial, sensory, and chemical properties of minced and raw meat stored for the period of 12 days at the temperature of 2°C (Boskovic et al. 2015). Therefore, it is now suggested that essential oils at various levels perform as an antimicrobial and antioxidant mediator, which in turn increase the shelf life of raw and minced meat. Higher oil concentrations are more effective than the lower ones for the safety and quality of meat and meat products (Salem‐Amany, Amine‐Reham, and Gehan 2010; Shaltout, Thabet, and Koura 2017).

## 8 Medicinal Applications

The main chemical ingredient of thyme essential oil is “thymol,” which actively demonstrates its effect against bacterial strains of *Staphylococcus* and *Salmonella*. Because of this activity, thyme becomes a useful aromatic plant with antiseptic characteristics in chronic fungal infections for the immune system along with another current therapy for respiratory tract infections, for example, whooping cough and bronchitis (Rašković et al. 2015). It can also be utilized as antiseptics, fumigants, mouth washes, and disinfectants. Minor chest and throat infections can be treated with pleasant tasting fresh thyme leaves by chewing, which may give relief against sore throat. Generally, thyme is recommended with other numerous medicinal herbs for the treatment of hay fever, worms, and asthma in children (Dauqan and Abdullah 2017).

Thymol, an active constituent of essential oil, shows its strong effect against coccid bacteria and enterobacteria. Thyme may also have the ability to improve liver function and act as a stimulant of appetite because thymol protects the microvilli action in stomach and increases the nutrients’ absorption that in turn increases the appetite and food intake as well as being useful for the treatment of inflammation and laryngitis (Saleh et al. 2014). Topical application of thyme is important in the sense that it can possibly be utilized for skin‐related problems, for example, acne, oily skin, bug bites, dermatitis (eczema), stings and also relieves severe pain due to damaged nerve anywhere in the body that may be caused by aging, diabetes, multiple sclerosis (disabling disease of brain and spinal cord), infection, pains, and rheumatic aches (Reddy et al. 2014) (Table 2).

**TABLE 2:** Uses of *Thymus vulgaris* in traditional medicine.

Medical properties	Plant part/plant preparation	Type of study	Place	Reference
*Traditional uses*				
Respiratory tract				
Cough, Asthma	Powder syrup	In vivo	Ardabil, Iran	Eskandarpour et al. (2024)
Pneumonia and Atelectasis	Essential oil	In vivo	Sari, Iran	Ghahremani Chabok et al. (2021)
SARS‐CoV‐2	Essential oil	In vivo	Zanjan, Iran	Sardari et al. (2021)
COVID‐19	Leaves dry powder	In vivo	Cameroon	Yiagnigni Mfopou et al. (2021)
Acute pneumonitis	Aerial parts	In vivo	Pécs, Hungary	Csikós et al. (2020)
Acute bronchitis	Dry extracts	In vitro/In vivo	Germany	Seibel et al. (2018)
Nervous system				
Analgesic activity/motor impairment	Aerial parts/thyme tincture and syrup	In vivo	Serbia	Rašković et al. (2021)
Alzheimer's disease	Essential oil	In vivo	Romania	Capatina et al. (2020)
Encephalomyelitis/Multiple sclerosis (MS)	Aerial parts	In vivo	Iran	Mahmoodi et al. (2019)
Cardiovascular system				
Hyperlipidemia	Leaves dry powder	In vivo	India	Ghai and Saraswat (2023)
Coronary angiogenesis	Not defined	In vivo	—	Roshankhah et al. (2020)
Hyperlipidemia	Leaves dry powder	In vivo	Iran	KoohiHosseinabadi et al. (2015)
Anxiety	Dry extracts	In vivo	Iran	Shaban et al. (2015)
Digestive system				
Gastrointestinal disorders	Leaves dry powder	In vivo	Tunisia	Rtibi et al. (2019)
Ulcer and ulcerative colitis	Essential oil	In vivo	Egypt	Hamed et al. (2022)
Intestinal diseases	Essential oil	In vivo	Peru	Rojas Armas et al. (2019)
Others				
Diuretic properties	Aerial parts	In vitro/In vivo	Romania	Babotă et al. (2023)
Emphysema	Essential oil	In vivo	Sari, Iran	Ghahremani Chabok et al. (2021)
COPD	Dry extracts	In vitro	Italy	Nabissi et al. (2018)

## 9 Pharmacological Activities

### 9.1 Antioxidant Properties

Thyme is one of the most important dried herbs from a group of analyzed herbal plants and contains a high concentration of total antioxidants (El‐Nekeety et al. 2011). Antioxidant level of thyme commercial samples was evaluated as more than 30‐fold in garlic and 2.8‐fold in ginger (Wei and Shibamoto 2010). Its essential oil has considerable antioxidant activity that can be measured in different assays including; nitric oxide, scavenging of 2, 2‐diphenyl‐1‐picrylhydrazyl radicals, suppression of peroxidation and lipoxygenase activities as well as inhibition of superoxide radical formation. Other antioxidant assays of essential oils include carboxylic acid assay, conjugated diene assay, and malondialdehyde (MDA) assay (Asbaghian et al. 2011).

Different case studies revealed the fact in this respect that essential oil of thyme is the most effective antioxidant among other oils, which were assessed earlier. The comparative study was conducted between thyme oil and the oils of rosemary, sage, basil, eucalyptus, cinnamon, chamomile, lavender, and clove. Experimental results of thyme oil and clove leaves demonstrated the considerable antioxidant activity and recognized as strong like α‐tocopherol and butylated hydroxytoluene (BHT) (Wei and Shibamoto 2010).

Thymol had been reported to scavenge ROS and control the antioxidant enzyme activity, which decrease the oxidative stress. The strong antioxidant property of thymol allows it to counteract ROS‐like superoxide anions (O_2_•−) and hydrogen peroxide (H_2_O_2_). Thymol also increases the important antioxidant enzyme activity that is essential for the detoxification of ROS, such as catalase (CAT), SOD, and glutathione peroxidase (GPx) (Ridaoui et al. 2022). Furthermore, it has been revealed that thymol transforms redox signaling pathways by adjusting the activity of kinases and transcription factors that are responsive to the redox state of cells. Consequently, thymol adjusts the way cells react to oxidative stress and activate defense mechanisms, which neutralize the harmful effect of ROS. Thymol gives the impression of a promising natural chemical for alleviating oxidative stress‐related disorders as well as improves general health through these mechanisms (Jan et al. 2024).

**FIGURE 3:**
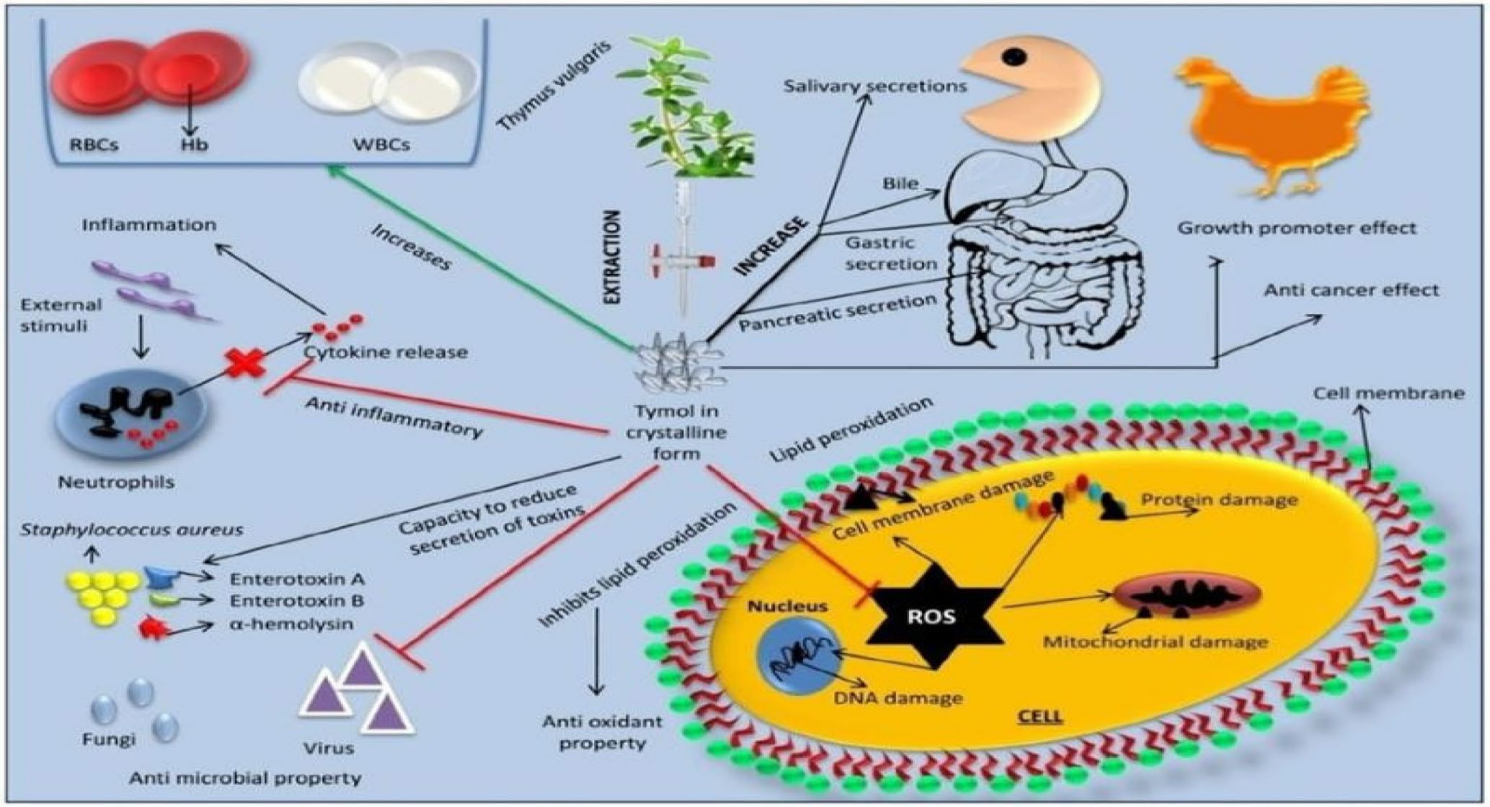
Pharmacological pathway of antioxidant, anti‐inflammatory, antimicrobial, and anticancer effect of thymol.

Owing to its effects on cells, thyme ethanolic extract utilized to pretreat HepG_2_ cells protected DNA from H_2_O_2_‐induced oxidative damage and encouraged the activity of GPx. Results after the analysis of ethanolic extract indicated that salvianolic acid (3.1 mg/g), luteolin hexoside (2.0 mg/g), apigenin glycosides (4 mg/g dry extract), and rosmarinic acid (14.7 mg/g) were the major biomarkers of antioxidant potential in a variety of tissues and due to its antioxidant studies, thyme can be used to treat chronic diseases most importantly, aging (Kozics et al. 2013). Mechanisms of action regarding the strong antioxidant potential are indefinite but include the regulation of signaling pathways and free radical scavenging, which control the production of endogenous antioxidant enzymes and molecules. More investigation is required to describe the effect of chronic and acute dietary intake of culinary thyme on biomarkers of oxidative stress in humans and animals (Asbaghian et al. 2011; Carlsen, Blomhoff, and Andersen 2011) (Figure 3).

### 9.2 Anti‐Inflammatory Activities

Preclinical evidence suggested that carvacrol (thyme constituent) encourages antiinflammatory effect and extracts of thyme have anti–inflammatory activities. For example, thyme oil repressed the activity of activated PPARα, COX‐2 promoter, and PPARFγ added in the concentration of 0.002%–0.008% to the endothelial cell culture (Hotta et al. 2010). Thyme oil was stated to contain 4% carvacrol, 6% p‐cymene, and 71% thymol when added to the cultures of oxidized low density lipoprotein‐stimulated THP‐1 macrophages; it significantly reduced gene expression and production of pro‐inflammatory intermediaries like tumor necrosis factor‐α (TNF‐α), IL‐6, and IL‐1β. On the other hand, it increased the gene expression and production of IL‐10 (Chan et al. 2005).

Thyme ethanolic extract when added to macrophage cultures (0.1%–0.4%) repressed the nitric oxide production and also decreased the inducible nitric oxide synthase mRNA expression, without varying the cell viability. However, methanolic extract of thyme was classified as the least potent among the eight spices in inhibiting nitric oxide release from lipopolysaccharide (LPS)‐activated macrophages (Amirghofran, Ahmadi, and Karimi 2012; Chohan et al. 2012). Studies about the effects of p‐cymene are not considered as important as those for thymol and carvacrol. Scientific studies must report the safety of chronic intake of culinary doses of thyme on biomarkers of inflammation in equally strong, healthy as well as sick animals with inflammation, to assess the capability of thyme to transform the equilibrium of anti‐inflammatory and pro‐inflammatory cytokines along with other signaling pathways in tissues (Vigo et al. 2004).

### 9.3 Immunomodulatory Effect

Thymol, a monoterpenoid that is obtained from thyme, validates remarkable immunomodulatory features, which have a strong impact on the adaptive and innate immune reactions. Research studies have proved that thymol stimulates phagocytosis, which is mainly essential for innate immunity and rises fluidity of the membrane, which sequentially increases macrophage action (Gierlikowska et al. 2021). It has been revealed that thymol upturns superoxide anion production by improving respirational eruption, through reducing the release of pro‐inflammatory cytokines that are IL‐1β and TNF‐α in LPS‐stimulated cells. This indicates that thymol functions as an anti‐inflammatory drug as it regulates cytokine production and inflammatory signaling pathways as well as immune cell function improvement (Gabbai‐Armelin et al. 2022).

In terms of adaptive immunity, thymol downregulates Th2 cytokines and transcription factors in allergic asthma, which drops airway hyperresponsiveness and inflammation (Mousa et al. 2021). Moreover, thymol function in reducing inflammatory reactions is further underscored by suppression of MAPK and NF‐κB signaling pathways. The therapeutic effects of thymol are related to immune‐mediated disorders and autoimmune diseases, as its immune‐stimulatory effects could be harnessed to reinstate immune balance in situations, which are characterized by dysregulated immune responses. On the whole, thymol shows promising agent as a natural therapy for immunological disorders and further research is required to determine the efficacy in clinical settings (Kowalczyk et al. 2020).

### 9.4 Antimicrobial Activity

Essential oils extracted from thyme leaves, which are being harvested at the fourth stage of biological process, were assessed for chemical composition and their biological activities. These explosive or volatile oils were investigated for the inhibitory action in contradiction of nine species of gram‐negative and six species of gram‐positive bacteria (Reddy et al. 2014). Plate counting technique was used to evaluate the inhibitory effect through direct contact, likewise the essential oil from thyme leaves is considered as the best operative remedy, which stops the development and growth of microorganisms (Reddy et al. 2017).

The mechanism of action involves the disruption of microbial cell membranes, which increases permeability and then cell lysis. Because of its lipophilic nature, thymol can participate into the lipid bilayer of bacterial membranes, which in turn changes the membrane integrity and causes structural damage. This breakdown affects the physical barrier of cells along with detrimental effects on cellular processes comprising production of energy and nutrition transport by hindering enzyme activities linked to membrane integrity and respiration (Cimino et al. 2021). Various studies have demonstrated that thymol can suppress the membrane‐bound enzyme activities, like H^+^‐ATPase, which are essential for maintaining the electrochemical gradient for ATP synthesis. This augments the antimicrobial efficiency of thymol against microorganisms like *Streptococcus iniae* and *E. coli* (Yin et al. 2022).

Thymol affects microbial gene expression and metabolic processes, in addition to cell membranes. Studies have reported that thymol exposure can amend the expression of genes linked with stress‐related reactions. The alteration in gene expression can make infections less able to survive ecological stressors, which would increase the efficacy of thymol. Thymol targets various microbial physiology elements, instead of depending on traditional antibiotic processes, which make it a practical technique for treating antimicrobial resistance (Yuan et al. 2018).

### 9.5 Antitussive Activity

Antitussive and spasmolytic action has been traditionally accredited to carvacrol and thymol (Basch et al. 2004). Through the inhibition of histamine receptors and acetylcholine, flavonoids in thyme appear to relax ileal and tracheal smooth muscles in animal models. In vitro utilization of extract and volatile oil of thyme applies soothing and relaxing effects on ileal as well as tracheal smooth muscles through the inhibition of contractions, which could as well be subjected to the presence of flavone aglycones (Gairola et al. 2010).

### 9.6 Anticancer Effect

Several compounds including the known flavanones were identified using different spectral techniques. Results of anticancer activity of thyme leaves extract in colorectal tumor cells exhibited the inhibition of proliferation process by following the time‐ and concentration‐dependent approach, which in addition contributes to anticancer activity; different bioactive compounds in thyme extract might have been proved to show the protective effect against human colorectal cancer (Ayesh, Abed, and Doa'a 2014).

The activation of apoptosis in cancer cells is one of the main mechanisms of thymol action. This is possible by ROS that cause cell cycle arrest at the G_0_/G_1_ phase and mitochondrial malfunction. Thymol had been revealed to give pro‐apoptotic properties and to alter important signaling pathways, which are linked to the advancement of cancer as it reduces the metastatic nodules in lung cancer models via obstructing the Wnt/β‐catenin pathway, which is important for metastasis and cell growth (Herrera‐Bravo et al. 2024). Moreover, it is also noteworthy that thymol interferes with signal transducer and activator of transcription 3 (STAT3) pathway. By blocking STAT3 phosphorylation, thymol inhibits downstream signaling, which supports proliferation and cell survival (Herrera‐Bravo et al. 2024). This results in a reduction of STAT3‐regulated genes, which include vascular endothelial growth factor (VEGF) and cyclin D_1_, which are essential for angiogenesis and tumor growth. Against the development of cancer, thymol had shown anti‐inflammatory and antioxidant properties that impart protective effect by lowering pro‐inflammatory cytokine levels (Zhang et al. 2020). The diverse effects of thymol on different molecular pathways highlight its potential as a beneficial agent in cancer treatment.

Cytotoxic effect of thyme extract toward head and neck cell carcinoma was investigated. Based on pharmacogenomics approach, novel perceptions about the molecular mode of anticancer activity of thyme are presented (Russo et al. 2015). The protective effect of the dry extract of thyme and its major active compound thymol against oxidative damage was evaluated. Thymol inhibited the generation or release of ROS in UV‐irradiated cells (Calò, Visone, and Marabini 2015).

### 9.7 Comparative Analysis of Thymol

Comparative studies of thymol have reported its advantages over conventional treatments in antimicrobial and anti‐inflammatory activities. It has greater anti‐inflammatory effect when used in combination with regular medicines like ketoprofen, which shows effectiveness in reducing edema in animals (Nagoor Meeran et al. 2017). Moreover, antioxidant properties of thymol contribute to its use as an adjunct therapy and offer safe alternative as compared to synthetic medicines. Synergistic effects have been shown when thymol is combined with commercial antibiotics, allowing reduced antibiotic doses and overcoming drug resistance (Gan et al. 2023). Thymol can be encapsulated as polymer in cellulose acetate and lignin derivatives that enable its controlled release and bioavailability, which resultantly enhances its antifungal and antibacterial activities. The pharmacokinetic differences between in vivo and in vitro studies require comprehensive research to optimize dose strategies and potential synergistic interactions with other drugs (Escobar et al. 2020). Overall, the integration of thymol in therapeutic procedures could pave the way for innovative cures in managing chronic inflammatory conditions and infections.

## 10 Potential Health Benefits

### 10.1 Effect on Respiratory Tract Infections (Asthma and Cough)

Asthma is an inflammatory disease associated with a strong oxidative stress that is a result of decreased antioxidant activity. It is a complex condition described by reversible airflow barrier and airway hypersensitivity associated with inflammation and a high level of Immunoglobulin E (IgE) antibodies is produced by the immune system in the case of allergy and the immune system overreacts to an allergen by producing the antibodies (Al‐Khalaf 2013). In traditional medicines, thyme possesses antispasmodic and broncholytic properties and is used in Central and Southern Europe for mitigation of mucosal inflammation of respiratory tract, bronchitis, and whooping cough (Singletary 2016).

Thymol and carvacrol have considerable effect on lungs, which provokes the secretion of mucous membrane and increases ciliary movement in bronchial epithelia (Al‐Khalaf 2013). In combination with other plant extracts, most human studies have estimated that thyme in a controlled trial like bronchipret tablets that contain ethanolic extracts of thyme and Primulae radix were compared with those of other herbal medicines like N‐acetylcysteine and Ambroxol for efficacy study of children and adults in treating cough. It was estimated that as compared with other reference medications, bronchipret provided a better therapeutic benefit (Singletary 2016). A placebo‐controlled and double‐blind research trial was being accomplished for the combined evaluation regarding the effectiveness of ivy extracts and thyme in adult patients with cough (Kemmerich, Eberhardt, and Stammer 2006). For this purpose, 351 patients of adult age were assigned to receive placebo or bronchipret treatment (35.4 mL/d) for 11 days. Effectiveness of this treatment is determined by self‐assessment of daily coughing fits, assessing the symptoms of cough and bronchitis. The group, which was given thyme–ivy extract, had substantial reduction of cough and faster bronchitis response rates than the placebo controls (Kemmerich, Eberhardt, and Stammer 2006). In vitro and in vivo data suggested that carvacrol in thyme is linked to bronchodilatory action. Efficacy of thymol could be investigated by a route of administration like; inhalation (nasal application) of thymol, which ensures a cough suppressant effect (Gavliakova et al. 2013).

Numerous results from clinical trials regarding thymol‐based treatments had been reported, which indicate both methodological issues and efficacy. Thymol's antibacterial and anti‐inflammatory properties had been studied regarding respiratory tract‐related diseases, particularly concerning chronic obstructive pulmonary disease (COPD) and asthma. When the concern is delivery techniques and dosage, studies about thymol suggest that it may support in improving lungs’ function as well as minimizing inflammation of airways (Baniamerian et al. 2023).

### 10.2 Gastrointestinal (GI) Tract Ailments

Carvacrol‐ and thyme‐rich water had been utilized in traditional medicines to relieve digestive tract ailments (Basch et al. 2004). The preclinical data provided an initial indication of GI tract benefits of thyme with different concentrations of thymol and carvacrol. Further research studies are needed to check the beneficial impact of thyme on the GI tract. Such calculations must include the evaluation of the effect of chronic dietary thyme intake at levels encountered in different food items used in gastrointestinal health (Mitsch et al. 2004). Thyme in powder form presented the inhibition in the growth of *S. typhimurium* when added to media and the effect of powdered thyme on the performance of broilers was studied, as it was found to have effect on the weight gain and improvement in the overall health of broilers, in addition to other behaviors, such as feed conversion ratio and feed intake. Because of these reasons, thyme plant is becoming more important due to its antimicrobial effect and the stimulating effect on human and animal digestive system (Al‐Kassie 2009).

### 10.3 Antinociceptive Effect

The antinociceptive (that inhibits nociception, the sensation of pain) and anesthetic (that induces insensitivity to pain) properties of thyme extract and its constituents are presented in in vitro and in vivo experimental designs. A randomized efficacy trial was conducted among 120 women who were suffering from painful menstruation (primary dysmenorrhea) (Direkvand‐Moghadam and Khosravi 2012). Women participants were divided into two groups; orally receiving either the treatment with ibuprofen (3 × 400 mg/d) or herbal treatment of Broncho T.D. that consists of essential oil of *Zataria multiflora* Boiss (Shirazi thyme) (4 × 5 mL/d). Broncho T.D. contains 1–1.5 mg thymol per 120 mL (Sajed, Sahebkar, and Iranshahi 2013). The most important outcome was severe menstrual pain measured by visual analog scale. Participants who were receiving ibuprofen (400 mg, 3 times a day) experienced reduction in pain that was not different from the pain reduced by orally administered herbal oil. This reduction in pain may be due to the result of antiprostaglandin (produces an anti‐inflammatory effect by inhibiting the formation of prostaglandins) and antispasmodic (suppresses muscle spasms) effects of herbal constituents (Iravani 2009).

### 10.4 Treatment of Neurological Disorders

Carvacrol shows multiple neuromodulatory activity, because of this reason it is used extensively as a food additive and has great probability for alleviating neurological disorders. By in vitro experiments it was revealed that α‐terpineol, thymol, carvacrol, and essential oil of thyme inhibited AChE activities. This has been proved with the fact that AChE inhibitors were developed for the cure of neurological conditions (Vladimir‐Knežević et al. 2014). Similarly, carvacrol exerted an AChE inhibitory effect, which is 10‐fold greater than that of thymol. It would be useful for animal models to be studied by using carvacrol‐containing thyme preparations in the diet to observe if at these levels, they control neurological issues and prevent brain damage. It could be helpful to assess thyme in combination with other plant‐based ingredients commercially available for the improvement of mood swings in humans (Zotti et al. 2013).

Clinical evidence in the context of nervous system‐related problems reported thymol's neuroprotective benefits, which had drawn attention predominantly to the perspective of its ability to reduce oxidative stress and inflammation in neurodegenerative disorders. Thymol might improve cognitive ability and reduce symptoms and occurrence of anxiety according to clinical trials. Though, the methodology and sample sizes which are used in these research findings are often variable, that promotes the questions about reproducibility and generalizability (Peng et al. 2024).

### 10.5 Benefits in Cardiovascular Diseases

Chronic inflammation, which is now considered as one of the most important reasons of the occurrence of cardiovascular diseases (CVDs), can be prevented by the incredible combination of anti‐inflammatory as well as antioxidant potential of thyme. As the antispasmodic effect of thyme is suggested to be responsible for the incidence of cardiac health, that's why its essential oil is predominantly advantageous regarding this aspect (Sharangi and Guha 2013). It allows the appropriate working of cardiac valves as well as unwinds or relaxes the arteries and veins, which in turn, strengthen the heart by reducing blood pressure. Hence, thyme is measured as a wonderful refresher or tonic, which improves the overall cardiac health, for example, it facilitates the proper and efficient functioning of heart (Estruch et al. 2013).

While concerning the design methodological considerations and outcomes of clinical studies, thymol had been investigated due to its ability to have cardioprotective effect on heart health and change lipid profile (El‐Marasy et al. 2020). However, the strength of the conclusion is restricted by methodological differences, such as sample size estimation, poor blinding, and randomization in various researches. For a variety of illnesses, a comprehensive analysis of clinical and preclinical research highlights the requirement for advanced methodological standards, which increase the rationality of inferences about the healing properties of thymol. On the whole, thymol exhibits potential as a therapeutic agent, but further advanced‐level clinical trials are necessary to confirm its efficacy and safety in treating respiratory, neurological, and cardiovascular disorders (Salehi et al. 2018).

### 10.6 Prevention From Iron Deficiency Anemia (IDA)

Thyme is an admirable nutritional commodity having various vitamins (vitamin A, vitamin C, riboflavin, and thiamine) and also minerals like zinc, calcium, magnesium, copper, potassium and most importantly; iron that fulfills the daily allowance of human body (Dauqan and Abdullah 2017). Iron is a natural element, which gives its vital role in numerous biochemical practices, including enzymatic reactions, electron transfer, and oxygen transport (Quintana et al. 2006). It is suggested as the most copious metallic element in brain and also contributes in major neurons‐related mechanisms, containing axon myelination and synthesis of neurotransmitter. It acts as the vital element for the increase of hemoglobin (Hb), energy production, and red blood cells. Iron deficiency might be able to cause tiredness, fatigue, increased susceptibility to infection, and anemia (Jonker and van Hensbroek 2014).

Some previous studies demonstrated the protective effect of thyme extract on blood serum biomarkers as well as on hematology that becomes the prevention strategy against IDA (El‐Sheikh 2008). In contradictory research, some experiments illustrated the point that thyme essential oil (polyphenols) gives inhibitory effect on the absorption of iron and decreases the blood iron concentration because of some bioactive compounds like saponins, tannins, and alkaloids and GI acidity that bind iron and give insoluble complex compounds in duodenal when released from thyme during digestion process (Ma et al. 2011; Gunshin et al. 2005). Synergistic effect of enough amounts of vitamin C and vitamin A in thyme enables its iron not to be bound, inhibits the formation of iron complexes with other antinutrients, and later gives its shielding potential against IDA and other blood disorders. The significant role of ascorbic acid for the absorption of nutritional or dietary non‐heme iron is acknowledged. The mechanism of action of vitamin C (ascorbic acid) can be described by two reasons that are; (1) ferric iron reduction to ferrous iron, which gives the impression to be a prerequisite for iron uptake into the mucosal cells and (2) prevention of the formation of insoluble and unabsorbable iron complexes (Beck et al. 2014).

#### 10.6.1 Absorption of Dietary Iron

The proximal jejunum and duodenum are the first two segments of small intestine where absorption of iron takes place as a result of the mechanisms that occur in the upper part of the GI tract (Fuqua, Vulpe, and Anderson 2012; Murata et al. 2021). As investigated earlier that a usual European food delivers around 14–15 mg of iron daily and from this, only 9%–10% of iron is absorbed in the intestine. On the other hand, the brush border of enterocytes becomes a site where specific iron absorption pathways have been reported as well as different import proteins of iron are also present in two ionic forms, for example, Fe^3+^ (ferric form) and Fe^2+^ (ferrous form); both of these are known as non‐heme iron molecules. There is also a storage protein (ferritin) with which non‐heme iron is associated. In the stomach, non‐heme iron at acidic pH becomes stable in its reduced form, that is, Fe^2+^ (Fuqua, Vulpe, and Anderson 2012). It is now becoming significant to notice that absorption of non‐heme iron is seized by a number of compounds, particularly plant polyphenols like tannins, saponins, and derived phytates. In this situation, non‐heme iron sourced from grains and vegetables can be absorbed by the synergistic action of organic acids like citric, ascorbic, malic acid, and others (Yersin et al. 2012).

Numerous pathways exist there, which support the absorption of non‐heme iron. Presently, there are numeral well‐organized guardian proteins present, which can be expressed in the enterocytes of duodenum and regulated in a different way as compared with the similar proteins in the cells of liver. Divalent metal transporter 1 (DMT1) is suggested as the best significant carrier of Fe^2+^ form (Rai et al. 2023b). Notably, ferric reductase enzyme activities because of the STEAPs (six transmembrane epithelial antigen of the prostate proteins) and duodenal cytochrome B, which are present on the brush boundary of duodenum, allow the reduction of Fe^3+^ to Fe^2+^ form of iron and consequently, facilitate the absorption of elemental iron through DMT1 (Choi et al. 2012).

#### 10.6.2 Transportation of Iron in the Blood

Transportation of iron in blood can be done mainly by the transferrin protein. Generally, the binding sites of the protein ranging between 20% and 40% are employed by ferric iron. Blood transportation is demonstrated to become a valuable factor, which is responsible for evaluating equally iron overload and iron deficiency. Transferrin (Tf) saturation or transportation is a sturdy sign of overloading of iron. Alternatively, as observed in blood transfused patients, the iron‐binding capacity of blood transferrin is repeatedly exhausted as considered from a physiological point of view, with associated production of non‐Tf‐bound iron (NTBI). By means of fluorescent marking out of labile iron in cytosol and endosomal vesicles, several explorations showed that NTBI elements are consequent from the sera of polytransfused thalassemia major patients, which can be capable of entrance via endocytosis into the cells (Sohn et al. 2011).

Erythrocyte (red blood cell) precursors take up iron restrictively via consuming transferrin, particularly transferrin 1, while other non‐erythroid cells and hepatocytes also use NTBI. Transferrin and iron bind to transferrin receptor (TfR) and these complex molecules are accepted in the cell by the recycling of vesicles of endosomes (Singh et al. 2024). Therefore, the cycle of Tf depends on the Tf–TfR complex trafficking, which involves internalization of the complexes in endosome, followed by release of iron, upon recycling of the Tf–TfR complex to the cell surface and acidification (decreasing the pH) of the endosomes that are membrane‐bound compartments inside the eukaryotic cells. Each of these steps is facilitated by a specific machinery and pathway (Chen and Paw 2012). Furthermore, TfR is cleaved or cut out and then sheds as a solvable form in intravascular space. The above‐mentioned shedding of TfR has been recognized for more than 30 years and assessment of it is established as a revealing sign of iron‐deficient erythropoiesis that gives information regarding the negative iron balance and cause exhaustion of iron stores that leads to IDA (Beutler, Hoffbrand, and Cook 2003; Zahn et al. 2013).

#### 10.6.3 Intracellular Iron Storage

The iron, which is transported to the mitochondria or cytoplasm, is Fe^3+^ and it is compulsory for Fe^3+^ to reduce into Fe^2+^ iron form. Likewise, DMT1 is also suggested as an important protein, which is involved in the transportation of iron from vacuole to the cytoplasm. Within the ferritin (iron storage) molecule, iron is associated with hydroxide anions and phosphate and stored in the Fe^3+^ form. Each and every ferritin molecule could be able to isolate up to around 4500 atoms of iron. It also has various enzymatic properties when iron is sequestered in the ferritin inorganic core and internalized as well as can convert Fe^3+^ iron to Fe^2+^ form. Some forms of ferritin are present in serum of human and can also be raised in the serious health conditions including iron overload and inflammation (Soe‐Lin et al. 2010).

When cellular ferritin production occurs in the virtual nonexistence of free cytosolic iron, secretion of ferritin occurs (De Domenico et al. 2011). There was an interesting point observed by a number of researchers who indicated that in macrophages, ferritin is not a significant source of iron for the metabolic activities of cells (Mikhael, Sheftel, and Ponka 2010). For many years, serum ferritin had been recommended for the assessment of maladies related to iron and its level or value has been reviewed recently as a sign of body's stored iron. Numerous genetic modifications in the genes of ferritin have been informed, which particularly relate with nervous system disorder (Koziol et al. 2001; Lehn et al. 2012).

## 11 Safety Issues of Thyme

Thyme and thyme oil are considered safe by the US (FDA) when used in food products as natural flavoring or seasoning agent. Though, thyme oil can be unsafe, when taken orally in undiluted form (Basch et al. 2004; Nieto, 2020). Carvacrol is one of the most extensive essential oil constituents, which is approved by FDA for utilization in food as an artificial flavoring agent and used in the permitted quantity to produce anticipated effect. It was included by the European Council in the list of flavoring ingredients that is called as B category, which means it could be added in food at the permitted level of 5 ppm for food, 2 ppm for beverages, and 25 ppm in candy. In this overview, data from animal efficacy studies presented the relevance of dose for culinary purpose (De Vincenzi et al. 2004).

Limited information regarding the toxicity of thymol, thyme, and carvacrol is available. Two percent or 10% thyme leaves diet was not poisonous for mice; the LD50 value of thyme essential oil in mice had been specified to be 4000 mg/kg (Fachini‐Queiroz et al. 2012). When analyzed in rats, the European Chemical Agency reported that thymol does not have long‐lasting adverse effects at 8–200 mg/kg dose, nor is it teratogenic at the dose of 67–667 mg/kg. In rats, thymol has an oral LD50 value of 980 mg/kg. For carvacrol, the oral LD50 value in rats is 810 mg/kg. Insignificant allergic reactions of external usage thyme products are possible in humans. No adverse effects have been observed from the intake of thyme herb combinations in human bronchitis studies (Jukic et al. 2007).

Data for phytochemicals of thyme, thymol, and carvacrol recommend that they have low toxicity for humans when used as spices for culinary items; on the other hand, the side effects, which are associated with chronic dietary thyme intake, need to be examined in animals, particularly when dietary thyme essential oils are considered to be used at concentrations greater than those used currently for food (Pelkonen, Abass, and Wiesner 2013). However, it is important to comprehend the dose‐dependent as well as toxic effect of thymol from the safety point of view. There are a lot of studies that explored the chronic and acute toxicity of thymol. In case of chronic toxicity, various research studies on mice exposed the inhalation of thymol for the duration of 6 months, at a dose which was 858 times higher than the maximum dose given to human in Epinephrine HFA, and did not report any toxic effect on lungs and respiratory system. The no‐observed‐adverse‐effect level (NOAEL) was measured to be 1.26 mg/kg/week for long‐term treatment and 0.42 mg/kg for single dose (Xie et al. 2019). On the other hand, acute toxicity studies reported that thymol has low toxicity profile, with an oral LD50 value of 980 mg/kg in rats, but higher dose might cause central nervous system depression and GI tract irritation (Nagoor Meeran et al. 2017).

Active thyme components may potentiate the general sedative effect, and other thyme constituents appear to interact with enzymes that metabolize the drugs and could intermingle with medications if taken in high amounts at the same time, and this is improbable at dietary level of consumption (Dong et al. 2012). One more concern, which is related to *Lamiaceae* family plants, is the presence of thujone, a bicyclic monoterpene ketone found in plants and used generally in food, herbal medicines, and beverages. Food product derivatives of the sage plant (*Salvia officinalis*), such as sage herbal tea, are the major contributors for its intake (Arceusz et al. 2013). Though, thyme has not been identified as a probable source of this compound and it has also not been detected in the essential oil of thyme (Amiri 2012).

## 12 Case–Control Studies of Thyme in Anemia

Minerals are important elements that have a significant role in different functions like growth, development, and maintenance of organs from building of strong bones and transmit nerve impulses in an individual organism. Iron is one of the most important elements in micronutrients as it produces hemoglobin, red blood cells and is important to treat IDA and produces myoglobin in muscles. Natural plants are good source of non‐heme iron and play their key role in treating anemia and IDA as well as used in recent studies because of the fact that they are “safe” to use and have well‐known applications in different experimental studies (Gupta 2014).

In an experimental study, Swiss albino rats (150–200 g weight) were distributed into four groups (six rats in each group) and the extracts of *Swertia chirata* leaves were administered by oral route daily for 4 weeks that reversed the incidence of anemia, which was potentially induced by phenyl hydrazine (PHZ). The mineral and vitamin composition of the leaves appear to be the most active constituents, which are accountable for the anti‐anemic activity of *S. chirata* leaves. These results support moderately the expected use of *S. chirata* in the treatment of IDA (Turaskar et al. 2013).

Another study having a 28‐day experimental period reported that there are totally four groups, three female and three male albino rats of Wistar strain in each group with 150–200 g weight were found. Group I served as normal control, group II as anemic control, group III as reference control, which is managed with vitamin B12, and group IV was treated with 200 mg/kg of ethanolic extract of leaves of *Kedrostis foetidissima*. All the experimental diets were orally directed just one time daily for the duration of 28 days. Blood was collected on Day 29, via rupturing of sinus with the help of phenobarbital anesthesia and then tested for Hb, red blood cells, and hematocrit estimation. The ethanolic leaf extract of *Kedrostis foetidissima* exhibited anti‐anemic activity against phenyhydrazine‐induced anemia in rats. The anti‐anemic effect produced by *K. foetidissima* leaves might have been showed due to its high content of iron, which is present in the plant (Baskaran and Suruthi 2016).

It has been demonstrated previously in an anti‐anemic study of *Spinacia oleracea* plant extract that was administered at 100 mg/kg body weight dose once daily to anemic Wistar rats of both sexes having 132–138 g weight during a 4‐week study period. The extract was found to be rich in phytochemicals, vitamins, and minerals that improved the body weight of anemic rats. The packed cell volume (PCV), Hb, and red blood cell level was increased significantly in *Spinacia oleracea‐*treated rats when compared with anemic untreated control group rats (Luka et al. 2014).

In severe toxicity trials, the lethal dose for 50% (LD50) of leaf extracts of *Thymus schimperi* was directed through everyday gastric ingestion for a period of 14 days to female rats of Wistar strain (five rats in each group). In this study, one control group and four experimental groups were used. Control group animals received only distilled water and level of doses, which were given to remaining groups, was 300, 2000, 5000, and 10,000 mg/kg for group I, group II, group III, and group IV, respectively. During this study, there was no observed sign of mortality and morbidity in female rats during the whole experimental period as well as no evidence of hepatotoxicity was recorded. The subchronic toxicity study of *T. schimperi* aqueous extract did not show any considerable effect on blood hematological parameters (Hb, red blood cells, MCV, hematocrit, MCH, and MCHC), liver and kidney, when administered at doses of 600 mg/kg and 200 mg/kg by oral route (Debelo et al. 2016).

In an experiment with 42 male Sprague–Dawley rats, they were provided with the diets containing thyme oil, extract, and powder groups for 8 weeks following the adaptation period of 1 week only. Thyme extract increased feed intake and body weight gain by thyme oil. As dry thyme (powder) proved to be rich in iron, iron intake and total iron absorption were highest for the rat groups fed on thyme powder. This dried powder and extract group diet showed a significant increase in packed cell volume (PCV), Hb, red blood cells, and glutathione (GSH) and significant decrease in MDA that alternatively gave hepatoprotective effect in IDA individuals as compared with control rat group. Hence, triglycerides and total lipids were decreased in thyme extract group followed by thyme powder and thyme oil diet (Al‐Badr 2011) (Table 3).

**TABLE 3:** The phytochemical composition of thyme and functional properties of the main components in thyme.

Phytochemical composition of thyme	Main Components	Functional properties	Reference
Phenolic acids	Vanillic acid	Preventive effect against Alzheimer's disease, antioxidant, antiviral, anti‐inflammatory, antibacterial, and immunostimulant properties.	Palmieri et al. (2020)
	Caffeic acid		
	Gallic acid		
	Syringic acid		
	Ferulic acid		
	Rosmarinic acid		
	p‐OH‐benzoic acid		
	Chlorogenic acid		
Essential oils	Carvacrol	Treatment of upper respiratory tract infections, symptoms of bronchitis, parasitic infections, and pruritus associated with dermatitis Possess antifungal, antioxidant, anti‐inflammatory, and spasmolytic activity	Vallverdú‐Queralt et al. (2014), Kowalczyk et al. (2020)
	Thymol		
	*p*‐Cymene		
	γ‐Terpinene		
	Linalool		
	β‐Myrcene		
	Terpinen‐4‐ol		
Flavonoids	Flavononols, Flavanone	Antioxidant, anti‐inflammatory, beneficial against chronic diseases in humans	Lorenzo et al. (2019) Palmieri et al. (2020) Mahrye et al. (2021)
	Methyl flavans Methyl flavones		
	Flavone glycosides		
	Apigenin		
	Luteolin		
	Quercetin		
	Myricetin		
	Kaempferol		
Biphenyl compounds	4′‐hydroxy‐5,5′‐diisopropyl‐2,2′‐dimethylbiphenyl‐3,4‐dione	Deodorant effect Antioxidant activity	Ladopoulou et al. (2015) Lorenzo et al. (2019)
	5,5′‐diisopropyl‐2,2′‐dimethylbiphenyl‐3,4,3′,4/‐tetraone		
	4,4′‐dihydroxy‐5,5′‐diisopropyl‐2,2′‐dimethylbiphenyl‐3,6‐dione		
	3,4,3′,4′‐tetrahydroxy‐5,5′‐diisopropyl‐2, 2′‐dimethylbiphenyl		
	3,4,4′‐trihydroxy‐5,5′‐diisopropyl‐2,2′‐dimethylbiphenyl		

## Authors Contribution

Muhammad Bilal Hussain designed the study and conducted the same under the supervision of Muhammad Afzaal and Farhan Saeed. Marwa Waheed and Muhammad Bilal Hussain performed the study and participated in drafting the article with Rushba Irfan. Farhan Saeed helped in developing the whole concept and editing. Noor Akram and Faiyaz Ahmed helped in preparing figures and tables, and the overall quality of the manuscript was maintained by Farhan Saeed. Gebremichael Gebremedhin Hailu, and Muhammad Afzaal wrote, edited, and revised the manuscript critically. The final version of the manuscript has been read and approved by all listed authors.

## Conflicts of Interest

The authors declare no conflicts of interest.

## Data Availability

Even though adequate data have been given in the form of tables and figures, however, all authors declare that if more data are required then the data will be provided on a request basis.
